# Overlaps of Skeletal Muscle Fatigue and Skeletal Muscle Damage: The Muscle Injury Continuum

**DOI:** 10.1186/s40798-025-00876-z

**Published:** 2025-06-10

**Authors:** Carsten Schwiete, Christian Roth, Joachim Mester, Holger Broich, Michael Behringer

**Affiliations:** 1 Department of Sports Sciences, Goethe University Frankfurt, Frankfurt, Germany; 2 German Research Centre of Elite Sport, German Sport University Cologne, Cologne, Germany; 3 Department of Performance, Neuroscience, Therapy, and Health, Medical School Hamburg, Hamburg, Germany

**Keywords:** Metabolic stress, Mechanical overload, Overlap, Tissue damage, Injury continuum

## Abstract

**Background:**

Muscle fatigue has long been identified as a potential risk factor for muscle overuse injuries, frequently occurring due to rapid eccentric contractions. Traditionally, muscle fatigue was thought to arise mainly to metabolic stress, whereas muscle damage was considered a consequence of mechanical overload. However, this binary approach fails to capture the complex physiological mechanisms, including prolonged-force depression, enzyme leakage or inflammatory responses, which overlap between both entities.

**Main Text:**

This narrative review synthesizes evidence regarding physiological and mechanical overlaps between muscle fatigue and muscle damage. It elaborates on the concept of a muscle injury continuum, including forms of muscle fatigue, possibly leading to mechanical tissue damage, and potentially culminating in severe muscle injuries. Additionally, the relevance of the overlaps for load monitoring and injury prevention in professional sports are discussed.

**Conclusion:**

Understanding and recognizing the interplay between muscle fatigue and muscle damage is crucial for developing individualized prevention strategies, minimizing injury risk, and enhancing performance. This comprehensive approach is vital for improving load management and ensuring the long-term health and efficiency of athletes.

## Key Points

- Muscle fatigue and muscle damage, traditionally viewed as separate entities, appear to overlap in physiological and mechanical processes.
- Fatigue and damage are stages within a larger muscle injury continuum, starting with physiological fatigue and ending in severe muscle injuries.
- A combination of laboratory-based studies and real-world observations underscore the complexity of these processes and reflect the need for careful monitoring, e.g. with selected biomarkers.

## Background

### Metabolic Muscle Fatigue and Mechanical Muscle Damage—An Outdated Concept?

Muscle fatigue is defined as a “[…] progressive decline of performance which largely recovers after a period of rest.” [1]. Historically, the prevailing belief was that the performance decline during muscle fatigue was primarily due to a shortage of available ATP to support energy-consuming processes during intense physical activity. However, muscle cells are equipped with effective countermeasures to prevent intramuscular ATP concentrations dropping into performance-affecting ranges [2]. Furthermore, it was assumed that under anaerobic conditions, lactate and hydrogen ions accumulate rapidly, leading to a drop in pH values and a malfunction of skeletal muscle cells. More recent data question not only the production of hydrogen ions as a result of lactate formation [3], but also the impact of low pH and high lactate concentrations on muscle function under physiological temperatures [4, 5]. A major driver of this “physiological fatigue” of the muscle is the accumulation of inorganic phosphate, which impairs Ca^2+^ release from and reuptake into the sarcoplasmic reticulum as well as Ca^2+^ sensitivity and maximal Ca^2+^ activated force [1].

In contrast to physiological fatigue, mechanical fatigue describes the development of musculoskeletal tissue damage in response to repeated mechanical stress, linked to a progressive performance decline [6]. When damage accumulates further in a specific tissue, it will ultimately reach its “fatigue limit” [7] and structural damage is caused. Interestingly, the resilience of different biological tissues, such as muscle tissue stiffness, can be altered through fluid changes among others [6]. Accordingly, the two main contributors to mechanical fatigue are the applied stress on the tissue and the mechanical properties of the respective tissue. If the applied force exceeds the tissue capacities, injury ultimately occurs. The relationship between the loading cycles and the tissue properties is commonly illustrated through the S–N (stress level vs. number of cycles to failure) curve [8]. The tissue damage can then be induced either due to a high number of low mechanical loads (high cycle loading) or due to a small number of high mechanical loads (low cycle loading) [6].

While mechanical fatigue is more often associated with high cycle loading (high number, low load), exercise-induced muscle damage (EIMD) is primarily linked high mechanical forces during unaccustomed eccentric exercises (low number, high load) [9]. The primary event leading to delayed-onset muscle soreness (DOMS) appears to be the mechanical disruption of the myofibrillar elements due to lengthening of the muscle fibers under load [10]. Eccentric exercise generates greater forces than isometric or concentric contractions [8, 9] due to a higher tension per cross-sectional area during contractions [11], imposing high mechanical stress on the contractile apparatus. This level of mechanical stress disrupts the myofibrillar ultrastructure at the Z-lines [12]. Despite being challenged [13], another proposed mechanism that leads to EIMD is addressed in the “popping sarcomere” theory [14], where the mechanical load during the lengthening contractions overstretches the weakest sarcomeres of the muscle fiber. Prolonged exercise increases the number of these “popped” sarcomeres [15], initiating subsequent microtears in the muscle and the symptoms of DOMS. These symptoms vary individually and include prolonged force deficits, muscle soreness, reduced joint range of motion, and enzyme leakage [16].

Conclusively, physiological fatigue has typically been attributed to metabolic stress, while mechanical fatigue and EIMD have been considered to result from different distinctions of mechanical overload. However, this distinction may not fully capture the complexity of the underlying mechanisms, given that muscle fatigue can also arise in the absence of metabolite accumulation [17] and EIMD is known to occur after exercise without high mechanical forces [15–17], supporting the principles of mechanical fatigue (high cycle loading).

This article outlines evidence that the two distinct yet interacting fatigue types significantly affect musculoskeletal tissue damage. A deeper understanding of the overlaps in fatigue and damage will help to understand injury processes, possible prevention strategies and has implications for rehabilitation. Therefore, we targeted searches in electronic databases (PubMed and Google Scholar) using keywords including “muscle fatigue”, “muscle damage”, “EIMD”, “DOMS” or “exercise physiology”. Articles were included if they (1) presented original research or well-accepted theoretical perspectives on muscle fatigue and muscle damage, (2) addressed experimental or applied settings to exercise performance or underlying cellular mechanisms, and (3) provided critical insights into possible overlaps of muscle fatigue and muscle damage.

## Main Text

### Overlaps of Muscle Fatigue and Muscle Damage

#### Prolonged Low-Frequency Force Depression

Even though physiological fatigue is usually short lived [20], fatiguing exercise at low frequencies results in prolonged force deficits, requiring several days to recover [21]. This delayed recovery, first described by Edwards et al. [21], is most evident after fatiguing exercise involving lengthening contractions [22]. In the past, physiological fatigue has often been linked to metabolic factors such as the accumulation of inorganic phosphate, reactive oxygen species or alterations of hydrogen ions [22, 23]. However, prolonged low-frequency force depression persists in the absence of significant metabolic changes [24]. Instead, mechanical stress resulting from eccentric contractions appears responsible for the decline in muscle performance, representing an overlap between fatiguing and damaging processes. The force deficits initially arise through constraints in the excitation–contraction coupling followed by a persistent deficit of contractile function, eventually impairing recovery [25]. This phenomenon of post-contractile depression has also been described by Allen et al. [1] and is linked to attenuated Ca^2+^ release from the sarcoplasmic reticulum and reduced myofibrillar Ca^2+^ sensitivity [26].

### Muscle Damage as a Result of Physiological and Mechanical Fatigue

During fatiguing exercise, muscle cell energy consumption can increase up to a 100-fold [21]. At the onset of physiological fatigue, there is an initial drop in force along with a concomitant rise of tetanic [Ca^2+^]_i_. The early force decrease is mainly associated with the accumulation of inorganic phosphate [P_i_], which hampers cross-bridge force. As the fatiguing exercise continues, tetanic [Ca^2+^]_i_ finally declines [1].

**Fig. 1:**
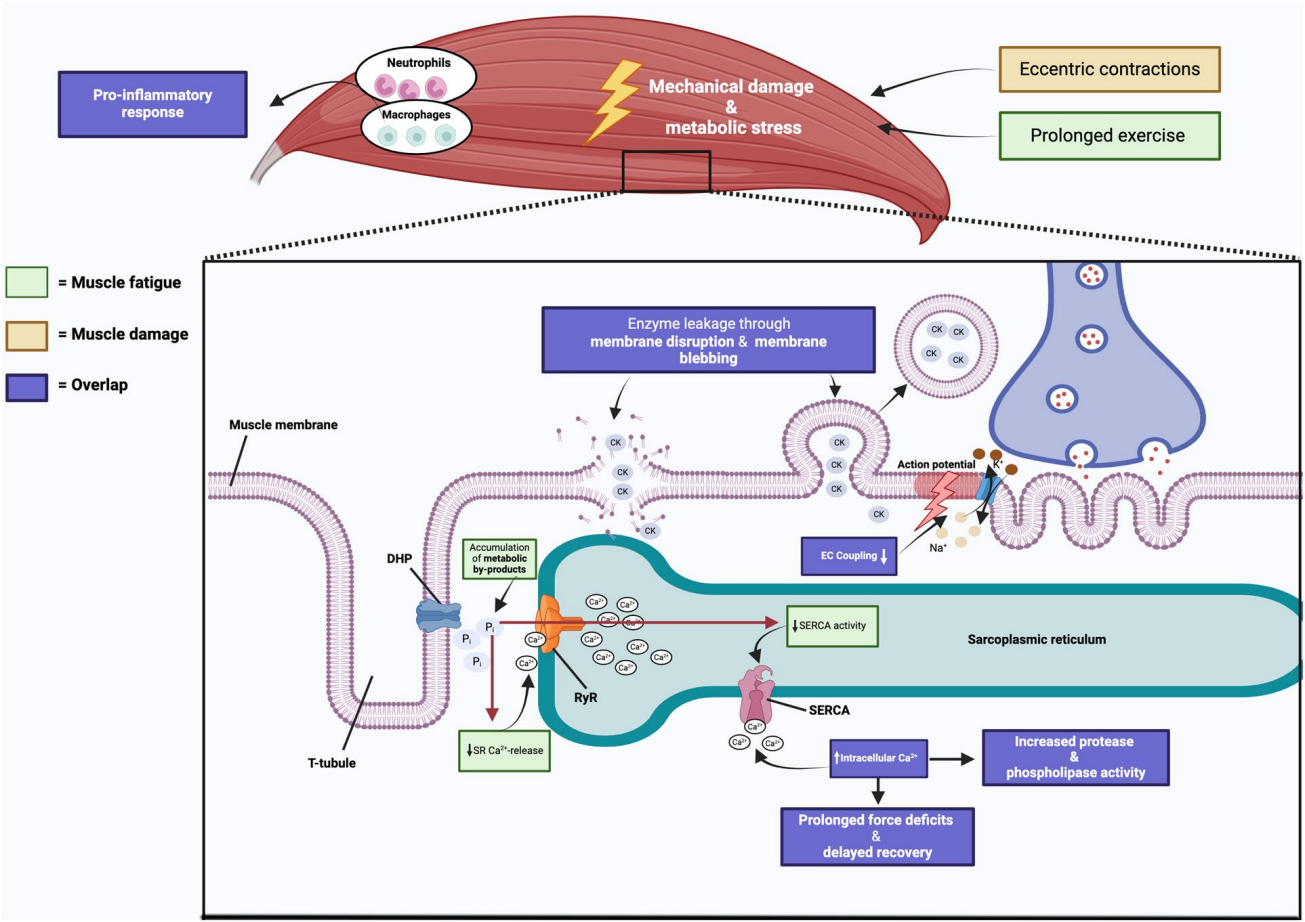
Overlapping physiological mechanisms of muscle fatigue and muscle damage. DHP = dihydropyridine receptor, RyR = ryanodine receptor, SERCA = sarcoplasmic/endoplasmic reticulum Ca^2+^-ATPase, SR = sarcoplasmic reticulum, EC = excitation–contraction, CK = creatine kinase, Na^+^ = sodium ion, K^+^ = potassium ion, Ca^2+^ = calcium ion. This image was created with BioRender

Despite the belief that eccentric contractions and high mechanical forces are the primary origins of EIMD, markers of muscle damage have also been reported after protocols without high mechanical forces [16, 17]. As mentioned, repetitive loading at lower stress levels (high cycle loading) can ultimately lead to tissue damage [6]. This form of mechanical fatigue represents another overlap in fatiguing and damaging exercise. For instance, after a single 3.5-h bout of intensive swimming in rats, creatine kinase (CK) levels peaked immediately after the exercise protocol and returned to baseline after 24 h [18]. The membrane damage therefore likely occurs independently from high mechanical tension as a result of increased metabolic stress. Amongst other processes, a rise in resting [Ca^2+^]_i_ during muscle damaging exercise is proposed [23]. During prolonged intense exercise, excitation-based Ca^2+^ influx may initiate muscle damage [27], with excessive Ca^2+^ accumulation activating Ca^2+^-dependent phospholipases and proteases capable of damaging the cytoskeleton (Fig. 1) [28]. Previous research on rats undergoing prolonged exercise indicated a ten-fold surge in Ca^2+^-uptake, with Ca^2+^ levels remaining elevated even after exercise [29]. This suggests progressive functional impairment and loss of membrane integrity, not necessarily dependent on high mechanical forces. Ultramarathon studies also demonstrate significant elevations in CK values and concomitant rise in muscle Ca^2+^, highlighting the prominent role of Ca^2+^ accumulation in muscle membrane damage [30].

Furthermore, Gleeson et al. [31] noted elevated blood lactate levels two days after EIMD, implying a shift towards greater dependence on non-oxidative metabolism. This metabolic aspect of DOMS has been attributed to enzyme leakage and metabolic exhaustion, initiating the breakdown of contractile tissue in the muscle [32]. Elevated levels of physiological fatigue during EIMD seem to impact the release of biomarkers associated with muscle damage. For example, unpublished data by Behringer et al. (2016) observed higher levels of CK and myoglobin following a downhill running regimen with the addition of blood-flow restriction compared to non-occluded controls.

### Inflammatory Responses

Both physiological fatigue and mechanical tissue damage elicit pro-inflammatory responses [33]. Exhausting endurance exercise and the associated fatigue leads to systemic releases of interleukin 6 and 8 and tumor necrosis factor-a (TNF-a), initiating neutrophile and monocyte recruitment [34]. TNF-a typically peaks as the primary cytokine after inflammation onset, followed by increases in interleukin 6 [35].

Notably, endurance events such as marathon running, which are known to elicit severe levels of DOMS, result in a greater inflammatory response than events with larger concentric components, such as cycling [36]. For instance, Suzuki et al. [34] reported increases of interleukin 6 up to a 100-fold after a marathon. Thus, the combination of metabolic stress and the degree of muscle damage during exercise appear to be key factors in cytokine release and neutrophil migration [36]. Further, these results underline the influence of the exercise’s contractile components on the post-exercise inflammatory response [35].

The levels of circulating inflammatory cells are key indicators of EIMD [37]. For example, leukocyte accumulation correlates with delayed muscle recovery after EIMD [38]. Neutrophils typically enter the inflammation site within the first 4 h, followed by macrophages during a second phase, approximately 24 h after muscle damage [39]. In severe cases of muscle damage, increased levels of leukocytes can persist up to 3 weeks post-exercise [40]. The amount of circulating leukocytes is also affected by the recruited muscle mass during exercise [41], explaining the strong inflammatory response after marathon running. In summary, the inflammatory response peaks when physiological fatigue and muscle damage coincide during exercise, indicating a potentiation of the two entities.

### Enzyme Leakage Can be Initiated by Mechanical or Metabolic Overload

One prominent symptom following EIMD is the accumulation of CK in the bloodstream [42]. Enzyme leakage after EIMD is primarily attributed to the greater mechanical tension during eccentric contractions, which often leads to myofibrillar damage and structural protein deterioration [16]. This loss of membrane integrity enables CK to move from the muscle tissue into the bloodstream [43].

While high mechanical tension is considered the main cause of enzyme expulsion, other studies have shown increased CK levels after endurance-based training protocols [15, 16, 43, 44]. Behringer et al. [28] proposed that the muscle cell’s energy status might influence enzyme release independent of mechanically-induced damage by ejecting CK “voluntarily” through membrane blebs to avoid cell death during extremely energy-demanding exercise. These fluid-filled membranous blebs, originally found in cardiac muscle cells during periods of hypoxia [45], may help the cell avoid necrosis. During prolonged exercise, the muscle cell may adopt these mechanisms, expelling ATP-consuming enzymes such as CK to protect itself.

Both metabolic stress and high mechanical forces lead to enzyme leakage from muscle cells. For example, CK and lactate dehydrogenase are enzymes that have been reported after mechanical damage and metabolic stress [30]. However, different time frames of CK increases have been observed. For instance, Overgaard et al. [30] reported CK peaking immediately after an ultramarathon, whereas CK typically peaks 2–3 days after high-tension eccentric exercise [46]. Additionally, Tee et al. [19] demonstrated that DOMS peaks 48 h following training programs with high mechanical forces. Conversely, DOMS peaks 24 h after exercise when significant metabolic stress is imposed [30]. This suggests that prolonged fatiguing exercise results in an earlier CK spike but also a quicker reduction of CK [28].

### Fatigue and Damage: Overlapping Phenomena?

Given the complexity of mechanisms in fatiguing and damaging exercise, it appears that physiological fatigue and mechanical damage might overlap in various scenarios. Based on the physiological overlaps such as attenuated Ca^2+^ release, CK-leakage or prolonged force depression, as well as the observed effects of fatigue on muscle strain injuries in elite sports [47], the two physiological scenarios appear closely intertwined and overlaps between the two processes have already been hypothesized by others [1, 18].

**Fig. 2:**
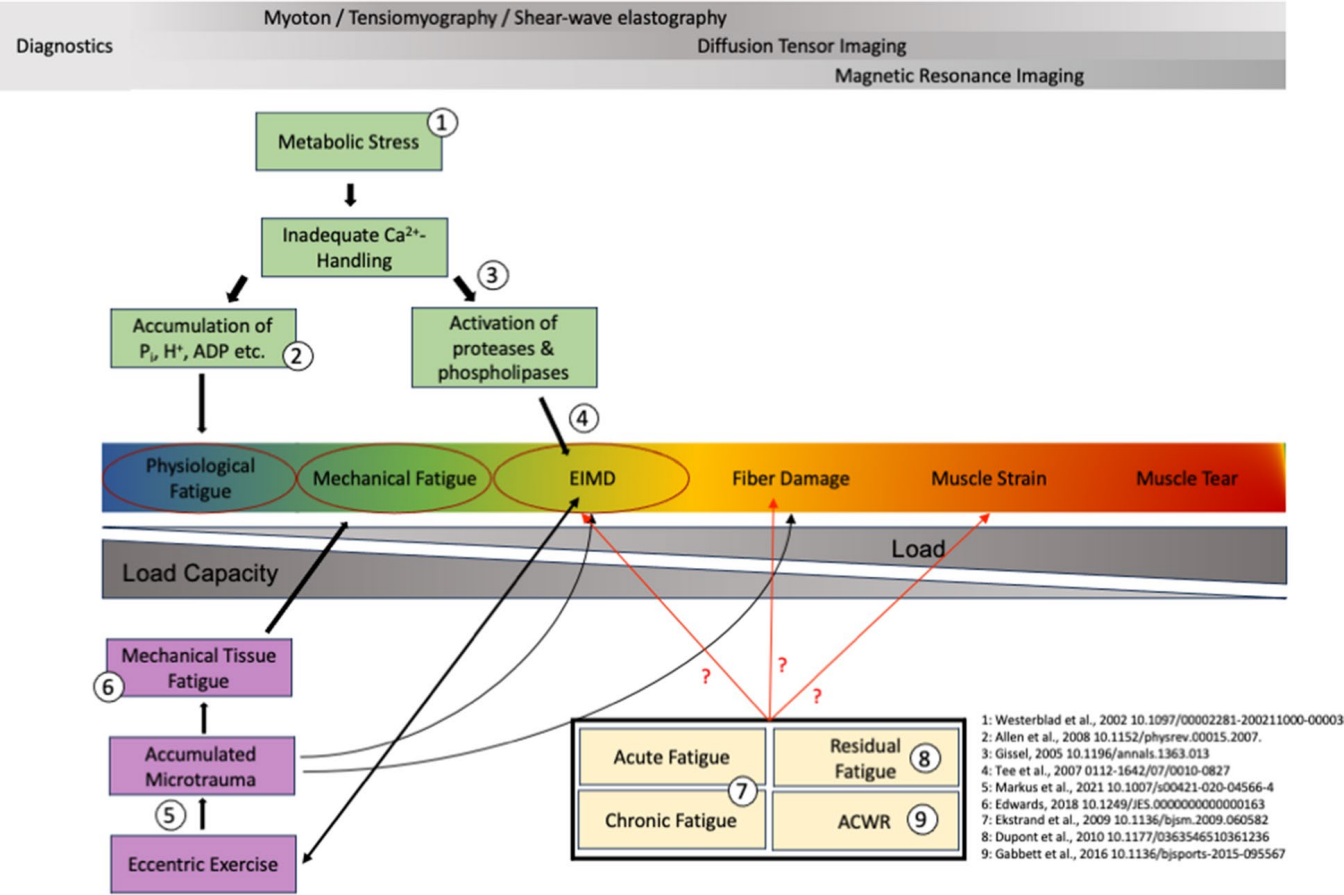
Illustration of the muscle fatigue continuum and its contributing factors. P_i_ = inorganic phosphate, H^+^ = hydrogen ion, ADP = adenosine diphosphate, Ca^2+^ = calcium ion, EIMD = exercise-induced muscle damage, ACWR = acute/cronic workload ratio

However, while the concept of an overlap is feasible, physiological fatigue and mechanical damage could also represent distinct gradations within a more extensive physiological continuum. Based on the existing literature, it is feasible that muscle fatigue and muscle damage are integral components within a broader physiological muscle injury continuum that unfolds in skeletal muscles as a response to strenuous exercise. Elaborating on the idea of Hyldahl and Hubal [49] that the muscle’s response to EIMD could act as a continuum, it is conceivable that these reactions represent only a fraction of a larger-scale continuum of physiological reactions. This muscle injury continuum may include a spectrum of responses that can culminate in mild to severe muscle damage, muscle strains, and even complete muscle tears (Fig. 2). The muscle’s “position” along this continuum is likely dictated by a multitude of related factors. For instance, different manifestations of muscle fatigue such as acute, residual, or chronic fatigue could possibly be viewed as the starting point on this muscle injury continuum. With ongoing load exposure and a concomitant decrease in load capacity due to muscle fatigue or damage, the muscle is moving further along the continuum.

The inability to reduce muscle injury incidence in professional sports over the past 18 years [48, 49] could be attributed to a misinterpretation of the athlete’s position along this muscle injury continuum.

### Physiological Fatigue and its Effect on Mechanical Damage

Based on the cellular mechanisms that are initiated by physiological fatigue and its temporal correlation with muscle injuries in various sports, muscle fatigue appears to be a universal starting point for further muscle damage. Muscle fatigue can have various causes such as neuromuscular impairments [52], metabolic changes [53], or mechanical wearing out due to eccentrically-induced tissue damage [54]. One of the central questions is how the physiological fatigue processes affect tissue damage in the forms of mechanical fatigue or EIMD. On the hand, physiological fatigue can represent a protective mechanism by decreasing mechanical load or shortening load exposure time. For example, during extensive sporting events, the onset of physiological fatigue might reduce the exposure time to repeated stress by limiting the amount of loading cycles. In addition, physiological fatigue is known to eventually lead to a force decline during subsequent contraction, that may lessen the mechanical load on the imposed tissue. In return, this might serve as a protective measure of the muscle to mitigate further structural damage. These effects have also been reported in laboratory-based settings, as Nosaka and Clarkson [46] showed that a concentric fatigue protocol performed prior to muscle damage attenuated the damage in the trained muscle. The authors concluded that the previous concentric exercise activated and prepared the muscle for the subsequent mechanical stress instead of increasing damage risk.

On the other hand, physiological fatigue can also increase tissue damage. First, the accumulation of metabolic by-products (P_i_, H^+^, ROS) reduces the muscle’s capacity to recover in between loading cycles, potentially favoring micro-damage progression. Second, fatigue is known to lead to changes in muscle fiber recruitment towards an increased fiber recruitment [55], resulting in further mechanical stress on fibers and increasing muscle damage susceptibility. Lastly, physiological fatigue can alter movement patterns and motor control. For instance, hamstrings injuries are suggested to occur primarily while running at maximum speed with fatigue leading to increased thigh flexion and maximum knee extension occurring later during the swing phase [56]. Because of these alterations, the hamstrings are then more excessively stretched.

Fatigue in real-world scenarios manifests in distinct types, encompassing acute, residual, or chronic fatigue. Although all these different types of fatigue appear to increase muscle injury risk, it is unclear whether the underlying mechanisms differ or whether they are simply different manifestations of fatigue. Among others, Gabbett et al. [53] illustrated the significance of the relationship between acute and chronic workloads and the associated fatigue. Acute fatigue is usually associated with metabolic acidosis [21] and neuromuscular inability to produce force [57], which increases the number of muscle injuries during a soccer match in the “UEFA study” by Ekstrand et al. [58]. Residual fatigue refers to the lingering fatigue effects over several days [59], decreasing performance [60]. A recent overview by Carmona et al. [61] has shown that 72 h between matches may not be sufficient in terms of recovery time for the hamstring muscle group. Chronic fatigue develops over time through repeated training sessions and inadequate muscle regeneration, leading to performance declines [62] and increased muscle injury risk [58]. While acute fatigue is mainly affected by metabolic and neuromuscular changes, residual and chronic fatigue are likely influenced by mechanical and tissue fatigue, bridging toward muscle damage on the muscle injury continuum. Mechanical fatigue is the accumulation of tissue damage in response to repetitive loading, linked to a progressive performance decline [6]. When damage accumulates further in a specific tissue, it will ultimately reach its “fatigue limit” [7] and structural damage is caused.

Both acute and chronic workloads and the athlete’s physical capacities dictate the trajectory along the continuum. If the load and the associated fatigue intensifies over days, weeks, and months, but the muscle’s load capacity decreases due to increased physical demands and limited recovery periods, the muscle might transition toward mild functional impairments and microtrauma emergence. If mechanical and metabolic loads increase further and the muscle cannot fully recover, the continuum’s far end will be reached, possibly resulting in substantial structural damage such as fiber tears or complete muscle tears. Accordingly, overuse injuries could ultimately be described as the result of mechanical fatigue of biological tissue [54], caused by repetitive bouts of lower-level stress [63]. This fatigue failure has been shown in animal models, where loaded tendons experienced damage progression at low but prolonged fatigue levels [64].

Similarly, Mueller-Wohlfahrt et al. [48] reported that functional muscle injuries could be attributed to fatigue (fatigue-induced muscle disorders, type 1A) or muscle damage (delayed-onset muscle soreness, type 1B). These muscle disorder types show no sign of structural alterations, but if not correctly evaluated and treated, they can act as precursors of further structural muscle injuries. For example, increased lower limb muscle tightness, a common functional muscle disorder symptom, is associated with a higher muscle lesion risk [65].

### Physiological Fatigue and its Effects on Muscle Injury Risk

Muscle injuries rank among the most common injuries in many sports, including soccer [55, 63], American Football [66], basketball [67], rugby [69], futsal [68], and badminton [70]. In top-level soccer, muscle injuries accounted for 40% of all injuries over the course of 18 consecutive seasons [50]. Likewise, muscle strains were the most common injury in the National Basketball Association (NBA) over the last 24 years, with a total of 1633 strains and 732 injured players [67]. The hamstrings have repeatedly been reported to be one of the most susceptible to injuries due to their biarticular nature and function during high-intensity, explosive running [71]. During the late swing phase of the running gait cycle, extensive hip flexion and knee extension excessively stretch the hamstring muscles under load, which appears to be the primary injury mechanism for this muscle group [72].

A recent meta-analysis by Maniar et al. [51] with over 7 million hours of exposure time has shown that the incidence of hamstring injuries has not changed over the last 30 years. In addition to other intrinsic injury risk factors such as age or injury history [73], muscle fatigue has been proposed as a major contributor to injury susceptibility for several years [46, 55, 71]. In the “UEFA” study by Ekstrand et al. [58], the authors found an increase in acute hamstring strains seemingly based on different types of fatigue. Acute physiological fatigue appeared to influence injury susceptibility as the number of injuries increases over match halves; in particular, hamstring injuries also increased with ongoing duration of the season due to an accumulation of chronic fatigue [58]. The latter is mainly attributed to the greater frequency and intensity of the games in this season phase, evoking residual fatigue in players lasting up to three days post-match [59]. Fatigue-induced injuries are also known from American Football, where the accumulated fatigue during the second and fourth quarter of the game is hypothesized to increase the number of injuries [66]. The prolonged NBA season and the associated accumulation of chronic and residual fatigue have been argued as a main factor for the increased number of muscle strains compared to college basketball [67], thereby supporting the findings of Ekstrand et al. [58]. Gabbett [75] reported a higher muscle injury rate in amateur rugby during the second half of the season (71%) compared to the first half (29%); in professional rugby, muscle injuries occur later in the training session and increase progressively towards the season’s end. These elite sports findings emphasize an important connection between muscle fatigue and muscle injury risk.

Although physiological fatigue appears to be a major risk factor for muscle strain injuries [47], the exact mechanisms behind these proposed effects are poorly understood. This can partially be explained by the complexity of investigating the overlap of both entities. In laboratory settings, EIMD is frequently employed as a controlled model to estimate possible effects on strain injuries as the EIMD-associated microtears are believed to be precursors of structural damage such as strains [73–75].

According to Friden and Lieber [77], a fatigued muscle is more prone to damage from consecutive lengthening contractions because the more fatigable fast fibers are stiffened and disrupted during the stretching process. Further, Mair et al. [78] reported that a fatigued muscle must be stretched further to absorb the same amount of energy compared to a non-fatigued muscle, ultimately increasing the risk for structural damage. This increased muscle stretching can significantly impact performance quality by affecting for example the running technique [79]. Next to these range-of-motion adaptations, reductions in eccentric hamstring strength after a soccer-specific fatigue protocol have been reported [80]. Interestingly, Greig and Siegler [81] found the largest decrements in eccentric hamstring strength at the highest movement speed, indicating a speed-dependent fatigue effect for eccentric hamstring strength. This speed-dependency might explain the increased number of injuries with prolonged match duration in soccer. As hamstring injuries mainly occur during high-speed running [80, 81], the fatigue effect on eccentric hamstring strength at high velocities appears to be a main contributor. Finally, Choi and Widrick [20] showed that a fatigue protocol preceding EIMD enhances immediate strength loss after eccentric exercise. These strength reductions appeared especially in eccentric contractions, offering another explanation for the increased number of injuries during muscle fatigue.

In contrast to these results, a substantial amount of laboratory investigations have not found a negative impact of muscle fatigue on EIMD. As mentioned, Nosaka and Clarkson [46] found protective effects of prior fatigue on subsequent EIMD. Similarly, Morgan et al. [14] revealed no differences in damage susceptibility between fatigued and non-fatigued muscles in cats and stated that EIMD is likely independent from muscle fatigue. A recent study from our own laboratory confirmed these results. In our investigation, neither acute nor residual physiological fatigue affected the subsequent amount of hamstring muscle damage in comparison to a non-fatigued control group [76].

Overall, it appears that a variety of laboratory-based investigations fail to confirm the effects of physiological fatigue on muscle damage, despite real-life observations suggesting otherwise. This illustrates the researchers’ difficulties in replicating the complexity of real-world conditions while working with highly standardized lab protocols and consciously manipulated variables. Even though these factors are essential for high-quality research, they may oversimplify the relationship between muscle fatigue, muscle damage, and consequent overuse injuries by overemphasizing or isolating selected variables. For instance, laboratory investigations might solely focus on concentric fatigue effects on eccentric muscle damage, while real-life muscle actions are indistinguishable and may vary in contraction velocity or muscle length.

In summary, muscle fatigue and muscle damage appear to be closely intertwined entities and part of a broader physiological muscle injury continuum. Muscle fatigue appears to be the continuum’s starting point, ultimately progressing to structural damage such as overuse injuries. Real-world observations consistently indicate a large impact of fatiguing scenarios and injuries. However, the exact mechanisms through which muscle fatigue contributes to these injuries remain complex and not fully understood. Laboratory investigations, while essential in research, often struggle to confirm these real-world scenario effects. The main difficulty lies in replicating the intricacies of the real-world conditions within highly standardized and isolated laboratory protocols. More high-quality research in cooperation with coaches and practitioners is needed to further close the gap between real-world observations and laboratory assessments.

## Implications for Practice

### Implications for Fatigue-Related Overuse Injuries and Load Monitoring

A deeper understanding of the interconnected physiological responses between muscle fatigue and muscle damage could benefit coaches and sports-medical practitioners in developing load monitoring strategies and injury prevention techniques. Overuse injuries occur when the mechanical load exceeds muscle load capacity and repetitive fatigue-related events likely reduce this load capacity. Therefore, investigating the precise interactions of muscle fatigue and muscle damage on muscle performance through high-quality research is crucial [20] while still aiming for an accurate replication of real-world situations. Considering that athletes in team sports exhibit a wide range of movement patterns and contraction types, including accelerations, decelerations, and changes of direction [82], replicating these situations in laboratory-based settings remains difficult.

The combination of pre-fatigued muscle and subsequent explosive eccentric contractions appears to be a major cause of overuse muscle injuries. Certain athletes seem to be more susceptible to specific exercise forms than others. Higher percentages of fast twitch muscle fibers can lead to greater fatigue levels, especially during maximal anaerobic activities due to increased metabolic demands. Among other factors, a possible mechanism for fatigue resistance differences may be lower inhibition of Ca^2+^ through intracellular Mg^2+^ in slow twitch fibers [83]. This raises the question of whether this is related to different fatigue resistance levels among athletes. High and low responses to EIMD have been identified based on parameters such as force loss [84] or CK response [83, 84]. However, there is still a lack of data on whether high and low responders to EIMD are related to fatigue resistance. Thus, an individualized approach based on a variety of physiological characteristics is essential in professional sports, as responses to muscle-damaging exercise vary significantly among athletes. Collecting comprehensive “physiological information” from participants or athletes, including systemic and peripheral metabolism or muscle-specific characteristics, e.g., skeletal muscle contractility or stiffness, can help determine why they respond in a specific way to muscle-demanding exercises. For example, unpublished data from our lab indicate that muscle fiber contraction time measured by tensiomyography can predict the amount of muscle damage 24 h post-exercise in the anterior thigh muscle.

Creating a holistic database of these parameters combined with precise response monitoring will aid in discerning whether an athlete’s reactions to a certain load are primarily due to muscle fatigue, muscle damage, or a combination of both. Identifying early signs of acute fatigue could assist in implementing proper recovery strategies to minimize the muscle damage response after two days. It is crucial for researchers and practitioners to know which blood markers are suitable for the defined goal of blood analysis regarding load management or health assessment. As emphasized by Haller et al. [86], the responsible sports medical professionals should analyze and interpret the collected blood markers considering within-subject changes, their overall experience with the individual athlete, and possible acute changes in other load parameters such as strength and or rate of perceived exertion. Additionally, practitioners should select biomarkers based on the physiological reactions of interest, as some markers are associated with muscle damage [87] and others with changes in the muscle cell’s energetic status [88].

### Biomarkers Within the Muscle Continuum

Accurately classifying the physiological and mechanical state of an athlete's muscle in the muscle injury continuum (e.g., physiological fatigue, EIMD etc.) is crucial for load management and injury prevention. In combination with subjective, internal and external load parameters, collecting blood markers can help practitioners interpret an athlete’s training status appropriately. To do so, collected blood markers must represent the accurate physiological “position” of the athlete on the muscle injury continuum.

While most blood markers are not exclusively associated with either muscle fatigue or muscle damage, several parameters show a strong correlation with one or the other. Lactate is probably the most popular blood marker for muscle fatigue, connected to high metabolic demands and glycolytic rate [86]. It reflects the muscle cell’s energetic situation as blood lactate increases as a sign of inadequate aerobic ATP generation [89], and accumulated lactate is highly correlated with the rate of perceived exertion [90]. Lactate accumulation is also related to a concomitant increase in hydrogen ions, known to reduce muscle pH and alter enzyme function after exercise. A direct assessment of hydrogen ions regarding fatigue state might therefore also be important, as they can interfere with Ca^2+^ handling or energy production [1]. Emerging biomarkers such as ammonia and hypoxanthine are often collected with lactate as indicators of muscle fatigue. Both are closely linked to the maintenance of the ATP:ADP ratio and inosine monophosphate degradation within the muscle cell [87, 89], indicating the muscle cell’s metabolic state. Another novel biomarker currently linked to muscle fatigue is the hormone irisin. While its exact releasing mechanisms are not fully understood, it is associated with changes in thermogenesis and glucose homeostasis in mice [91]. Acute exercise bouts appear to elevate irisin levels [92] while chronic training leads to decreased circulating irisin [91]. Although these biomarkers may serve as potential tools to assess an athlete’s physiological status more accurately in the future, significant methodological challenges need to be solved before routine implementation in everyday practice is realistic.

Regarding muscle damage, CK is commonly used as a biomarker to estimate the degree of muscle damage after unaccustomed exercise and for load management regarding daily training in professional sports [86]. However, some data indicate that blood CK can also increase in the absence of mechanical stress [28] and substantial individual differences have led to the description of high and low responder types [85]. Given the complexity in interpreting CK levels concerning actual muscle damage, there is ambiguity regarding its reliability as a muscle damage marker [91–93].

Apart from its usage as a damage marker, CK serves a critical function in energy metabolism. Often described as the muscle cell’s energetic regulator, CK assists in maintaining adequate ATP levels to sustain ongoing muscle contractions by facilitating the conversion of creatine and ADP into creatine phosphate and ATP [94]. This suggests that increased energetic demands for the muscle can result in a naturally elevated CK turnover rate, even in the absence of overt muscle damage. Considering the wide variability in CK levels following muscle damage and its substantial role in energy metabolism, its exclusive use as a pure damage marker might not be ideal in the sports medical field. Due to its large molecular size, CK enters and clears the blood circulation slowly. Therefore, more rapidly cleared molecules with a smaller size such as myoglobin may be used as an alternative. Myoglobin levels have been reported to increase after a soccer match [95] and mixed martial arts fights [96], indicating their usefulness as an acute, indirect marker for muscle damage.

In summary, utilizing blood markers to identify an athlete’s position on the muscle continuum is complex and requires a variety of parameters. Further in-depth knowledge about the physiological reactions and their overlaps is needed to interpret the results accurately.

## Conclusion

This narrative review outlines the intertwined relationship between muscle fatigue and muscle damage, possibly positioning them as interconnected stages along a broader muscle injury continuum. The traditional view that separates metabolic muscle fatigue and mechanical muscle damage into distinct categories fails to capture the complexity of physiological responses observed in both laboratory and real-world sports settings. Instead, evidence suggests that these processes overlap and may sequentially contribute to the development of more severe muscle injuries. This continuum model has significant implications for injury prevention and load management. Recognizing the early signs of muscle fatigue and implementing strategies to mitigate its progression could be critical in preventing the transition to more severe muscle damage and overuse injuries. This approach necessitates a shift towards individualized monitoring techniques that consider the unique physiological responses of each athlete, particularly in high-stress environments such as professional sports. Advanced biomarker analysis and precise load monitoring could offer valuable tools in this regard, enabling practitioners to tailor training and recovery protocols more effectively.

## Abbreviations

ATP: Adenosine triphosphate
pH: Potential of hydrogen
Ca^2+^: Calcium
EIMD: Exercise-induced muscle damage
DOMS: Delayed-onset muscle soreness
P_i_: Inorganic phosphate
CK: Creatine kinase
TNF-⍺: Tumor necrosis factor alpha
Mg^2+^: Magnesium
ADP: Adenosine diphosphate
